# Lemierre’s Syndrome With External Jugular Vein Thrombosis Following Submandibular Sialadenitis: A Life‐Threatening Complication

**DOI:** 10.1155/crot/4595472

**Published:** 2026-09-02

**Authors:** Sara Torretta, Lorenzo Cavaleri, Lorenzo Gaini

**Affiliations:** ^1^ Fondazione IRCCS Ca’ Granda Ospedale Maggiore Policlinico, Milan, Lombardy, Italy, policlinico.mi.it; ^2^ Università Degli Studi di Milano Dipartimento di Scienze Cliniche e di Comunità, Milan, Lombardy, Italy

**Keywords:** external jugular vein thrombosis, *Fusobacterium necrophorum*, Lemierre’s syndrome, septic pulmonary emboli, submandibular sialadenitis

## Abstract

**Background:**

Lemierre’s syndrome is a rare but potentially life‐threatening condition traditionally characterized by bacteremia, septic thrombophlebitis of the internal jugular vein, and metastatic septic emboli following pharyngotonsillitis. We report an atypical presentation originating from submandibular sialadenitis and complicated by external jugular vein thrombophlebitis.

**Case Presentation:**

A 26‐year‐old man presented with high‐grade fever, sore throat, and right submandibular swelling persisting for 7 days. Contrast‐enhanced CT revealed right submandibular sialadenitis, thrombosis of the right external jugular vein without abscess formation, and bilateral cavitary pulmonary lesions consistent with septic emboli. Blood cultures identified *Fusobacterium necrophorum* as the causative pathogen. The patient was managed in the intensive care unit with vasopressor support, high‐flow nasal oxygen, weight‐based therapeutic anticoagulation (enoxaparin), and targeted intravenous antibiotics. Complete clinical and radiological recovery, including venous recanalization, was achieved without surgical intervention.

**Conclusion:**

Clinicians must maintain a high index of suspicion for Lemierre’s syndrome even when thrombosis involves noninternal jugular neck veins or when the primary infectious focus is nonpharyngeal. Prompt multidisciplinary management is key to favorable outcomes.

## 1. Introduction

Lemierre’s syndrome, historically termed postanginal septicemia or necrobacillosis, is a severe septicemic illness predominantly affecting young, previously healthy adults [1]. First systematically described by André Lemierre in 1936, the classical syndrome comprises an acute oropharyngeal infection, septic thrombophlebitis of the internal jugular vein (IJV), and metastatic septic embolization, most commonly to the lungs [2]. The annual incidence is estimated at 3–6 cases per million, with a peak in adolescents and young adults aged 14–24 years and a male predominance [1]. The primary pathogen is *Fusobacterium necrophorum*, an anaerobic Gram‐negative bacillus known for its potent endotoxins, leukotoxins, and hemagglutinins that facilitate mucosal invasion, tissue destruction, and hypercoagulability [3, 4]. Less frequently, other anaerobes, *Fusobacterium nucleatum*, *Streptococcus* species, and *Staphylococcus aureus* are isolated [3, 4]. While classical Lemierre’s syndrome follows pharyngotonsillitis or peritonsillar abscess with subsequent spread to the parapharyngeal space and IJV, atypical anatomical variants are increasingly recognized [4, 5]. Diagnostic workup relies on contrast‐enhanced computed tomography (CT) of the neck and chest, which provides definitive visualization of venous thrombosis and pulmonary septic emboli [4]. Management mandates prolonged antimicrobial therapy (typically 3–6 weeks) including agents active against anaerobic organisms and *β*‐lactamase‐producing bacteria (e.g., metronidazole, *β*‐lactam/*β*‐lactamase inhibitor combinations, or third‐generation cephalosporins) [4, 6]. Anticoagulation therapy may be considered in selected cases to reduce the risk of septic embolism, despite the lack of robust evidence supporting its efficacy [5, 6]. In this article, we describe an atypical case of Lemierre’s syndrome presenting with submandibular sialadenitis and external jugular vein (EJV) thrombophlebitis and septic pulmonary emboli, successfully managed with conservative medical treatment.

## 2. Case Presentation

A 26‐year‐old man with no relevant medical history (weight: 80 kg) presented to the emergency department (ED) with severe right submandibular swelling, neck tenderness, and persistent high‐grade fever (> 38.5°C). The onset of neck discomfort began 7 days prior to ED presentation; 4 days after symptom onset, he was treated with outpatient oral azithromycin (500 mg daily) and prednisone (25 mg daily) by his primary care physician but experienced progressive clinical deterioration over the subsequent 3 days. Physical examination revealed an acutely ill patient with severe swelling in the right submandibular region, with overlying erythematous edema. Additionally, a firm cord‐like structure was palpable in the right lateral cervical region corresponding to the course of the EJV. Oral examination showed normal dentition and mucosal structures. Manual expression of the submandibular gland did not result in purulent discharge from Wharton’s duct. Laboratory tests revealed elevated inflammatory markers, with a C‐reactive protein (CRP) level of 323.6 mg/L (normal range: < 5.0 mg/L) and a white blood cell count of 26,160/μL (normal range: 4000–10,000/μL), predominantly neutrophils (absolute count 24.69 × 10^3^/μL, 94.5%).

**FIGURE 1:**
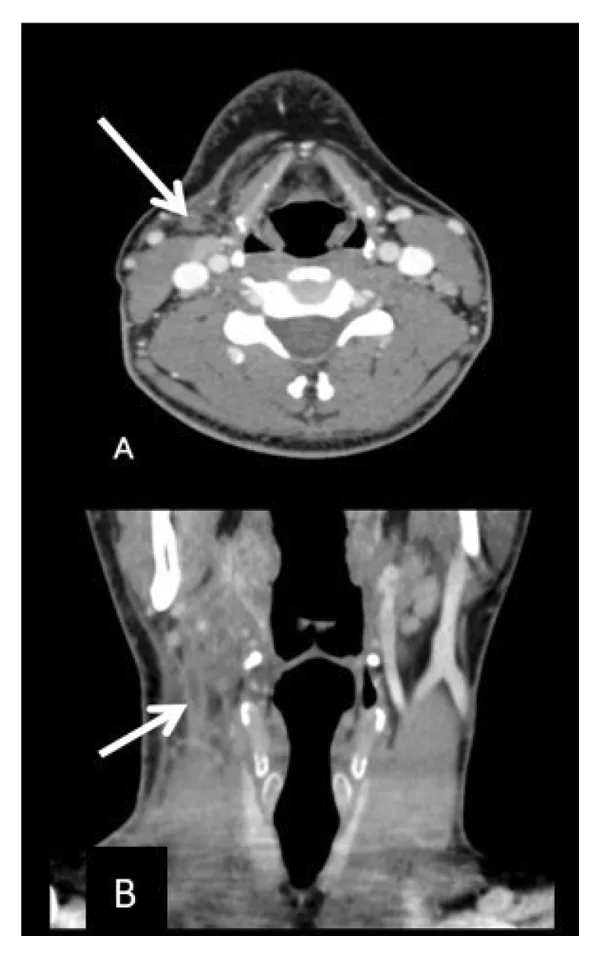
Contrast‐enhanced CT images of the neck showing right submandibular gland swelling and thrombosis of the right external jugular vein in axial (A) and in coronal (B) view.

Emergency neck ultrasonography demonstrated enlargement of the right submandibular gland, along with the presence of an incompressible, hyperechoic cervical vein, consistent with venous thrombosis. A contrast‐enhanced CT scan of the neck revealed diffuse enlargement, edema, and heterogeneous enhancement of the right submandibular gland, with soft‐tissue fat stranding in the surrounding tissues, without evidence of abscess (Figure 1). A filling defect was noted within a venous structure extending along the right EJV (Figure 1).

**FIGURE 2:**
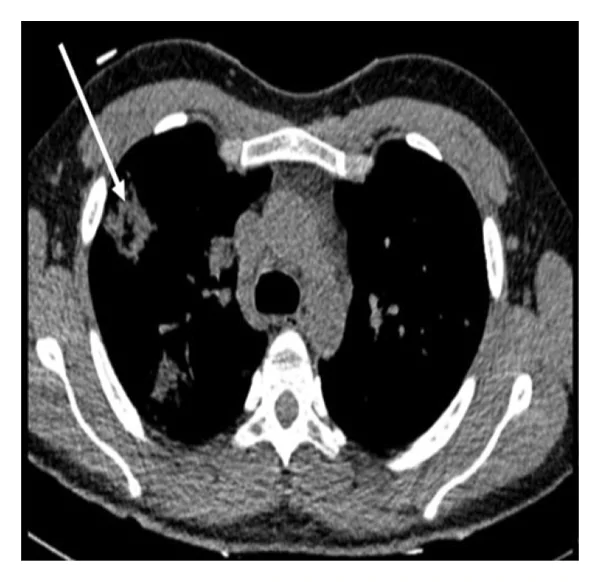
Contrast‐enhanced chest CT image showing multiple bilateral cavitary pulmonary lesions consistent with septic emboli.

Due to the onset and worsening of tachypnea and hypoxia, a contrast‐enhanced chest CT scan was obtained. The CT revealed multiple bilateral multilobar parenchymal lesions with cavitary features, consistent with septic pulmonary emboli and micro‐abscesses (Figure 2).

The patient was subsequently transferred to the intensive care unit (ICU) for severe sepsis/septic shock. Hemodynamic stabilization required fluid resuscitation and low‐dose vasopressor support (norepinephrine up to 0.08 μg/kg/min for 36 h). A high‐flow nasal cannula (50 L/min, FiO2 0.40) was initiated; invasive mechanical ventilation was not required. Empirical intravenous antibiotic therapy was initiated with a continuous infusion of piperacillin–tazobactam (18 g/day), in combination with linezolid (600 mg twice daily) and clindamycin (600 mg four times daily). Weight‐based therapeutic anticoagulation with enoxaparin (8000 IU [80 mg, 1 mg/kg] twice daily) was administered.

Baseline blood cultures yielded *Fusobacterium necrophorum*. Based on the culture results and antibiotic susceptibility testing, the antimicrobial regimen was de‐escalated to IV cefotaxime (2 g QID) and IV metronidazole (500 mg TID). Brain magnetic resonance imaging (MRI) was performed in the ICU to evaluate potential central nervous system embolization, which was negative for acute ischemic or infectious foci. The case was discussed in a multidisciplinary meeting with otolaryngology specialists, who did not recommend surgical intervention due to the patient’s favorable clinical evolution. Repeat blood cultures drawn on hospital Day 4 and day 7 were sterile.

Due to the patient’s progressively improving clinical condition in the ICU, he was transferred to the infectious diseases ward after 10 days for continued monitoring and treatment. The clinical course remained favorable, with progressive improvement in inflammatory markers, respiratory function, and radiological findings. The patient was subsequently discharged from the hospital on Day 18 with ongoing anticoagulant therapy (enoxaparin 6000 IU twice daily stepped down during transition to direct oral anticoagulants) and a 6‐week course of outpatient antibiotic therapy consisting of ceftriaxone (2 g daily) and metronidazole (500 mg three times daily).

At 3‐month and 6‐month postdischarge follow‐ups, the patient remained fully asymptomatic. Follow‐up Doppler ultrasonography and chest CT demonstrated complete resolution of the pulmonary lesions and full recanalization of the right EJV.

## 3. Discussion

Classical Lemierre’s syndrome typically arises from oropharyngeal infection involving the IJV. In our patient, the infection originated from submandibular sialadenitis rather than the more typical oropharyngeal focus, with progression to thrombophlebitis of the EJV instead of the IJV. Primary salivary gland infection is an exceptionally rare trigger for Lemierre’s syndrome, with fewer than 30 cases of EJV‐associated Lemierre’s‐like syndrome reported in the medical literature across both adult and pediatric populations [5, 7, 8]. Anatomically, retrograde or local contiguous venous drainage from an inflamed submandibular gland into the facial vein and tributaries can communicate directly with the EJV, bypassing the primary IJV system [7].

Moreover, no primary abscess formation was observed, either clinically or radiologically, further distinguishing this case from the classic presentation of Lemierre’s syndrome and underscoring that septic thrombophlebitis can proceed directly from localized parenchymal inflammation.

Surgical intervention (e.g., incision and drainage, EJV/IJV ligation, or excision) was historically common, but contemporary management favors conservative medical therapy [4, 6]. Ligation is now reserved for refractory septic embolization or persistent bacteremia despite adequate antimicrobial cover [6]. In our patient, early aggressive medical management yielded prompt clinical clearance without surgical intervention.

The role of anticoagulation therapy remains debated due to a lack of randomized controlled trials. Some authors suggest its use in patients with extensive thrombosis or persistent bacteremia to reduce the risk of further septic embolization [6, 9]. In our case, weight‐based therapeutic anticoagulation with enoxaparin (1 mg/kg twice daily) was started early and continued during hospitalization and postdischarge, with good clinical outcomes and complete recanalization of the EJV at 3 months follow‐up without bleeding complications.

*Fusobacterium necrophorum* remains the quintessential pathogen due to its unique virulent repertoire, including leukotoxins and platelet‐aggregating hemagglutinins that directly promote intravascular thrombosis [3, 4]. While traditionally considered susceptible to penicillin, increasing reports of β‐lactamase‐producing strains mandate the inclusion of β‐lactamase inhibitors or metronidazole, which exhibits superior tissue penetration and bactericidal activity [4, 6].

### 3.1. Clinical Implications

This case highlights that Lemierre’s syndrome should not be ruled out based on the absence of pharyngotonsillitis or IJV involvement. Clinicians must maintain a high index of suspicion for Lemierre’s syndrome whenever any cervical venous thrombosis (including EJV, facial, or retromandibular veins) coexists with systemic sepsis, elevated inflammatory markers, or pulmonary cavitary lesions in young adults presenting with head and neck infections.

## 4. Conclusion

Atypical presentations of Lemierre’s syndrome involving EJV thrombosis and nonpharyngeal primary foci, such as submandibular sialadenitis, present diagnostic challenges. Prompt contrast‐enhanced imaging, early microbiological blood cultures, targeted anaerobic antimicrobial therapy, and weight‐based therapeutic anticoagulation are key to achieving complete recovery without invasive surgical intervention.

## Author Contributions

Sara Torretta: clinical management and manuscript drafting and revision.

Lorenzo Cavaleri: clinical management and critical revision of manuscript.

Lorenzo Gaini: literature review and visual data/figure preparation.

## Funding

Publication fees are covered by 2026 Ricerca Corrente grant from the Italian Ministry of Health.

## Disclosure

All authors read and approved the final manuscript.

## Ethics Statement

The authors have nothing to report.

## Consent

Written informed consent was obtained from the patient for publication of this case report and accompanying images.

## Conflicts of Interest

The authors declare no conflicts of interest.

## Data Availability

The data supporting the findings of this study are available from the corresponding author upon reasonable request.
